# BRCA1 Expression is an Important Biomarker for Chemosensitivity: Suppression of BRCA1 Increases the Apoptosis via Up-regulation of p53 and p21 During Cisplatin Treatment in Ovarian Cancer Cells

**Published:** 2007-03-02

**Authors:** Akiko Horiuchi, Cuiju Wang, Norihiko Kikuchi, Ryosuke Osada, Toshio Nikaido, Ikuo Konishi

**Affiliations:** 1 Department of Obstetrics and Gynecology, Shinshu University School of Medicine, 3-1-1 Asahi, Matsumoto 390-8621, Japan; 2 Department of Regenerative Medicine, Toyama University Graduate School of Medicine and Pharmaceutical Sciences, 2630 Sugitani, Toyama 930-0194, Japan

**Keywords:** BRCA1, ovarian cancer, biomarker, chemosensitivity, cisplatin

## Abstract

BRCA1 is a tumor suppressor which plays a crucial role in the repair of DNA double-strand breaks, and its abnormality is responsible for hereditary ovarian cancer syndrome. It has recently been reported that reduced expression of BRCA1 is also common in sporadic ovarian carcinoma via its promoter hypermethylation, and that ovarian carcinoma patients negative for BRCA1 expression showed favorable prognosis. To address if BRCA1 expression plays a role in the chemotherapeutic response, we analyzed the effect of BRCA1 suppression on the sensitivity to cisplatin and paclitaxel in ovarian cancer cells. Specific siRNA for BRCA1 gene was transfected into 3 ovarian cancer cell lines with various p53 status. Reduced expression of BRCA1 by transfection of BRCA1-siRNA resulted in a 5.3-fold increase in sensitivity to cisplatin in p53-wild A2780 cells, but not in p53-mutated A2780/CDDP and p53-deleted SKOV3 cells. Regarding the sensitivity to paclitaxel, BRCA1 suppression caused no significant changes in all the 3 cell lines. For ionizing radiation sensitivity, BRCA1 suppression also showed a significant higher sensitivity in A2780 cells. Growth curve and cell cycle analyses showed no significant differences between BRCA1-siRNA-transfected A2780 cells and control cells. However, cisplatin treatment under suppression of BRCA1 showed a significantly increased apoptosis along with up-regulation of p53 and p21 in A2780 cells. Accordingly, reduced expression of BRCA1 enhances the cisplatin sensitivity and apoptosis via up-regulation of p53 and p21, but does not affect the paclitaxel sensitivity. Expression of BRCA1 might be an important biomarker for cisplatin resistance in ovarian carcinoma.

## Introduction

Epithelial ovarian carcinoma is the leading cause of death from gynecological cancer (Ozols et al. 2000). BRCA1 is a tumor suppressor gene responsible for hereditary ovarian cancer syndrome (Miki et al. 1994), and has been shown to regulate the maintenance of genome integrity, cell cycle control, apoptosis and DNA repair (Wang et al. 2000; Scully et al. 2000). Although somatic mutation of the BRCA1 gene has rarely been detected (Futreal et al. 1994; Merajver et al. 1995; Berchuck et al. 1998), decreased expression of the BRCA1 mRNA and protein (Thompson et al. 1995; Zheng et al. 2000; Russell et al. 2000), allelic loss or loss of heterozygosity (LOH), and methylation of the BRCA1 promoter region have recently been reported in sporadic ovarian carcinomas (Catteau et al. 1999; Esteller et al. 2000; Baldwin et al. 2000; Geisler et al. 2002; Wang et al. 2004). In addition, several studies have reported better survival in patients with hereditary BRCA1-associated ovarian carcinoma compared with those with sporadic carcinoma (Boyd et al. 2000; Ben David et al. 2002; Rubin 1996). Even among sporadic ovarian carcinomas, we showed that patients with advanced ovarian carcinoma negative for BRCA1 expression tended to show better survival than those with carcinoma positive for BRCA1 (Wang et al. 2004). These findings suggest that patients with BRCA1-negative carcinoma respond well to chemotherapy. To explore the role of BRCA1 gene in the response to chemotherapy, we analyzed the effect of BRCA1 suppression by BRCA1-specific siRNA on the sensitivity to anti-cancer agents such as cisplatin and paclitaxel, as well as to ionizing radiation in ovarian cancer cells with different p53 status.

## Materials and Methods

### Ovarian cancer cells

The ovarian cancer cell lines SKOV3 and OVCAR3 were purchased from the ATCC (Rockville, MD). The ovarian cancer cell lines A2780 and A2780/CDDP (a cisplatin-resistant cell line derived from A2780) were kind gifts from Dr. Takashi Tsuruo (Cancer Chemotherapy Center, Tokyo, Japan)(Tsuruo et al. 1986) with the permission of Dr. Thomas C. Hamilton (Fox Chase Cancer Institute, Philadelphia, PA). A2780 and A2780/CDDP were maintained in PRMI 1640 (Sigma, St. Louis, MO) supplemented with 10% fetal bovine serum (FBS) (Biomeda, Foster City, CA). SKOV3 was cultured in Dulbecco’s modified Eagle’s medium (DMEM) (Sigma) with 10% FBS. Incubation was carried out at 37°C under 5% CO_2_ in air.

### siRNA and transfection

BRCA1 siRNA sequences were identified using the Dharmacon website (Dharmacon Research, Lafayette, CO). SI20 (UGC ACU AGC CUC ACA CAU AdTdT), a scrambled version of SI20 (SCR) (CCU ACU AAG CGA CAC CAU UdTdT), or the control luciferase siRNA (UAAGGCUAUGAAGAGAUACdTdT) were used in this study. Ovarian cancer cells were transfected with siRNA duplexes by using Oligofectamine (Invitrogen Carisbad CA), following the manufacturer’s instructions.

### RNA Extraction and RT-PCR

Total RNA was extracted by the acid guanidinium-phenol-chloroform method as described previously (Horiuchi et al. 2003). One microgram of total RNA was treated with 1 U/10 *μ*l DNase I (Life Technologies, Gaithersburg, MD).

Reverse transcription and polymerase chain reaction (RT) was performed using an RNA PCR Kit (Takara Shuzo, Otsu, Japan), the 1 *μ*g RNA sample being added to 20 *μ*l of a reaction mixture consisting of 10 mM Tris-HCl (pH 8.3), 50 mM KCl, 5 mM MgCl_2_, 1 mM dNTP mixture, 1 unit/*μ*l of RNase inhibitor, 0.25 units/*μ*l of avian myeloblastosis virus-derived reverse transcriptase, and 0.125 *μ*M of oligo d(T)-adaptor primer. Using a thermal cycler (Perkin Elmer, Gene Amp PCR System 2400-R, Norwalk, CT), the reaction mixture was incubated at 42°C for 30 minutes, heated at 99°C for 5 minutes, and then cooled down to 5°C for 5 minutes.

One microliter of the RT products, containing 50 ng reverse transcribed total RNA, was amplificated by adding 20 *μ*l of PCR reaction mixture containing 10 mM Tris-HCl (pH 8.3), 50 mM KCl, 2.5 units/100 *μ*l of TaKaRa Taq DNA polymerase, with 0.2 *μ*M of a set of oligonucleotide primers. Primers were synthesized to encompass a specific segment of the cDNA sequence of the BRCA1, (sense, 5′-TGAGGCATCAGT CTGAAAGCC-3′ and antisense, 5′-CTGAT GTGCTTTGTTCTGGA-3′), or of G3PDH (glyceraldehyde-3-phosphate dehydrogenase) (sense, 5′-ACGACCACTTTGTCAAGCTC-3′ and antisense 5′-TCACA GTTGCCATGTAGACC-3′, spanning between exons 7 and 8). The corresponding cDNA fragments were denatured at 94°C for 30 seconds, annealed at 58°C for 1 minute, and extended at 72°C for 1 minute. After 35 cycles of amplification, the PCR products were analyzed on a 2% agarose gel, and the bands were visualized using ethidium bromide during exposure to an ultraviolet transilluminator.

### Drug sensitivity assay

WST-1 assay was employed for the assessment of drug sensitivity. SKOV3, A2780 and A2780/CDDP cells were seeded onto 96-well tissue culture plates at a density of 10,000 cells/well. After 24 hours, cells were incubated in medium supplemented with the described concentration ranges of cisplatin (Sigma) and paclitaxel (Bristol Myers Squibb). After 72 hours of continuous drug exposure, cultured cells were incubated with WST-1 reagent (Roche, Indianapolis, IN) at a dilution of 1:10 in the original conditioned media for 4 hours. After thorough shaking, the formazan produced by the metabolically active cells in each sample was measured at a wavelength of 450 nm with Multi-scan JX (Thermo Labsystems, Vantee, Finland). Absorbance readings were normalized against control wells with medium alone.

### Cell cycle analysis

We used FACScan for the cell-cycle analysis. Forty-eight hours after siRNA transfection, each group of cells was collected and washed with PBS(−) three times. The cells were fixed in 70% ethanol. Then, the cells were resuspended in a DNA-stain solution containing propidium iodide (20 mg/ml; Calbiochem, CA, U.S.A) and RNAase (1.8 units/ml; Sigma, St. Louis, MO, U.S.A). The cells were analyzed with a FACScan (Fluorescence Activated Cell Sorter) flow cytometer equipped with an argon laser (488 nm; Becton Dickinson Immunocytometry System, Mountain View, CA, U.S.A). The experiments were repeated three times.

### Western blot analysis

Cells were lysed in a lysis buffer: 50 mM Tris-HCl, pH 8.0, 0.25 M NaCl, 0.5% NP-40, 1 mM PMSF (Sigma), 1 *μ*g/ml aprotinin (Boehringer Mannheim, Germany), 1 *μ*g/ml leupeptin (Boehringer Mannheim), and 20 *μ*g/ml TPCK (Boehringer Mannheim). The lysates were centrifuged at 13,000 rpm for 20 minutes at 4°C and the supernatants were stored at −80°C. Extracts equivalent to 50 *μ*g of total protein were separated by SDS-polyacrylamide gel electrophoresis (8% acrylamide) and transferred onto nitrocellulose membranes (Hybond TM-C super, Amersham, U.K.). The membranes were blocked in TBST (0.2M NaCl, 10mM Tris, pH 7.4, 0.2% Tween-20) containing 5% non-fat dry milk and 0.02% NaN3 for 1 hour, then incubated with the first antibodies in TBST containing 5% non-fat dry milk. The membranes were then incubated with the sheep anti-mouse or rabbit Ig (Amersham) in TBST containing 2% non-fat dry milk. Bound antibody was detected with an enhanced chemiluminescence system (Amersham). The density of bands was quantified by densitometric analysis using a Quantity One Scan System (ATTO, Tokyo, Japan).

Antibodies used for Western blotting were the rabbit polyclonal, BRCA1 antibody, D-20 (Santa Cruz Biotechnology), ERK, pERK (Cell Signaling Technology, Inc. Danvers, MA), p53, p21, Bax, BCL-2 (Santa Cruz, St Louis, MO), and *β*-actin (Biomakor, Rehovot, Israel). For PARP assays, we used the monoclonal antibody #556494 (BD Bioscience. San Jose, CA U.S.A), which specifically recognizes the full-length *M*_r_ 116,000 PARP protein and its *M*_r_ 85,000 and 25,000 cleaved products.

### Apoptosis assay

Quantitation of apoptotic cells was performed after 72 hours of continuous drug exposure, using ApoStrand ELISA Apoptosis Detection Kit (BIOMOL International, LP) according to the manufacturer’s instructions. The ApoStrand ELISA is based on formamide denaturation and the detection of the denatured DNA with an antibody to single-stranded DNA. Absorbance was defined at 405 nm (Thermo Labsystems, Vantee, Finland).

### Irradiation experiment

Approximately 48 hours after siRNA transfection, cells were either left untreated or exposed to 3 and 6 Gy of ionizing radiation (Irradiation Equipment MBR-1505R2, Hitachi Medical corp., Tokyo, Japan).

### Statistical analysis

The data are presented as the mean±SD. The significance of differences was assessed by the Kruskal-Wallis test or by Mann-Whitney’s U test. Differences were considered to be significant when P<0.05. These analyses were made using the Stat-View system (Abacus, Berkeley, CA, U.S.A).

## Results

### BRCA1 is expressed in all of ovarian cancer cell lines

**Figure 1:**
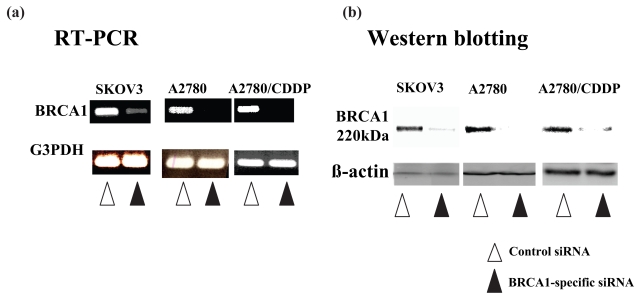
Ovarian cancer cells express BRCA1 mRNA and protein, which is suppressed by BRCA1-specific siRNA transfection. RT-PCR (a) and Western blot (b) analyses show that all of the 3 ovarian cancer cell lines, SKOV3, A2780 and A2780/CDDP, express endendogenous BRCA1 mRNA and protein. Transfection of the BRCA1-specific siRNA suppresses the expression of BRCA1 in all of the 3 cell lines. Lanes 1, SKOV3; 2, A2780; 3, A2780/CDDP.

All of the 3 ovarian cancer cell lines, SKOV3, A2780 and A2780/CDDP, expressed endogenous BRCA1. Inhibition of endogenous BRCA1 expression after transfection with the BRCA1-siRNA oligonucleotide was confirmed by RT-PCR and Western blot analyses. BRCA1 expression of these BRCA1-siRNA-transfected cells was decreased, compared with that in the same cell lines transfected with the control oligonucleotide (Fig. 1).

### Suppression of BRCA1 enhances the sensitivity to cisplatin in p53-wild ovarian cancer cells

**Figure 2:**
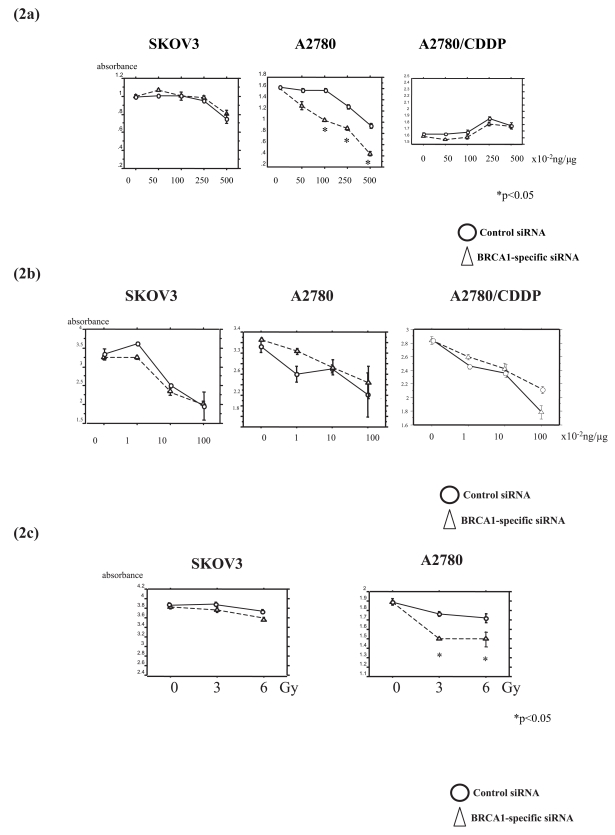
Suppression of BRCA1 expression results in the increase in the cisplatin sensitivity in A2780 ovarian cancer cells. WST-1 assay shows that effect of cisplatin (a), paxlitaxol (b), and irradiation (c) on the viable cell number. Transfection of BRCA1-siRNA results in the increase in the sensitivity to cisplatin in p53-wild A2780 cells, but not in p53-mutated A2780/CDDP or in p53-deleted SKOV3 cells. Sensitivity to paclitaxel is not changed in all of the 3 cell lines. Values indicate means ±SE. Significance of differences from the control is * P<0.05, ** P<0.01.

We examined the role of BRCA1 expression in the sensitivity to chemotherapeutic agents in the presence or absence of endogenous BRCA1 expression. After 3 days of drug exposure at a concentration of 50, 100, 250, or 500×10^−2^ ng/*μ*g of cisplatin, the number of cells was decreased under cisplatin treatment in a dose-dependent manner in the control-siRNA-transfected A2780 cells: to 81% of control with 250×10^−2^ ng/*μ*g and to 69% of control with 500×10^−2^ ng/*μ*g cisplatin (Fig. 2a). BRCA1-siRNA transfection resulted in a significant decrease in the viable cell number of A2780 cells under cisplatin treatment: to 65% of control with 100 × 10^−2^ ng/*μ*g, to 56% of control with 250 × 10^−2^ ng/*μ*g, and to 26% of control with 500 × 10^−2^ ng/*μ*g cisplatin. Accordingly, BRCA1-siRNA-transfected A2780 cells displayed a >5.3-fold increase in sensitiveness to cisplatin compared with the control cells. In contrast, SKOV3 and A2780/CDDP cells did not show the change in the sensitivity to cisplatin by BRCA1-siRNA transfection (Fig. 2a).

### Suppression of BRCA1 does not change the sensitivity to paclitaxel, but enhances the irradiation effect in ovarian cancer cells

Sensitivity to paclitaxel was also examined in all of the 3 cell lines after inhibition of endogenous BRCA1 using BRCA1-siRNA. Although A2780 cells showed a slight increase in the resistance to paclitaxel by transfection of BRCA1-siRNA, all of the 3 cell lines did not show significant changes in the sensitivity to paclitaxel (Fig. 2b).

Next, we examined the sensitivity to ionizing radiation. Exposure to 3 and 6Gy irradiation resulted in a significant decrease in the number of viable A2780 cells after BRCA1-siRNA transfection compared to that of control cells (Fig. 2c). However, this effect was not observed in other cells.

### Suppression of BRCA1 does not affect the cell cycle of ovarian cancer cells

**Figure 3:**
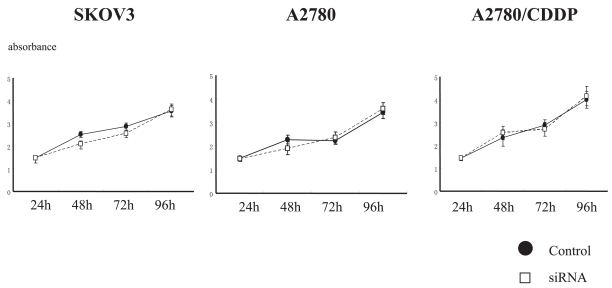
Suppression of BRCA1 expression does not affect the cell proliferation of ovarian cancer cells. There is no significant differences in the growth curves between BRCA1-siRNA- transfected cells and control-oligonucleotide-transfected cells. Values indicate means ±SE.

To investigate whether the increase in chemosensitivity by suppression of BRCA1 expression depends on the cell proliferation, we examined the effect of BRCA1-siRNA transfection on the cell growth of ovarian cancer cell lines. Growth curves using WST-1 assay did not show the significant difference between BRCA1-siRNA-transfected cells and the control cells in all of the 3 cell lines (Fig. 3). The cell-cycle analysis by FACScan showed that there was no significant difference in the fractions, such as G1, S, and G2/M phases, between BRCA1-siRNA-transfected cells and the control cells in all of the 3 cell lines (Table 1).

### Suppression of BRCA1 results in an increase of apoptosis under cisplatin treatment

**Figure 4:**
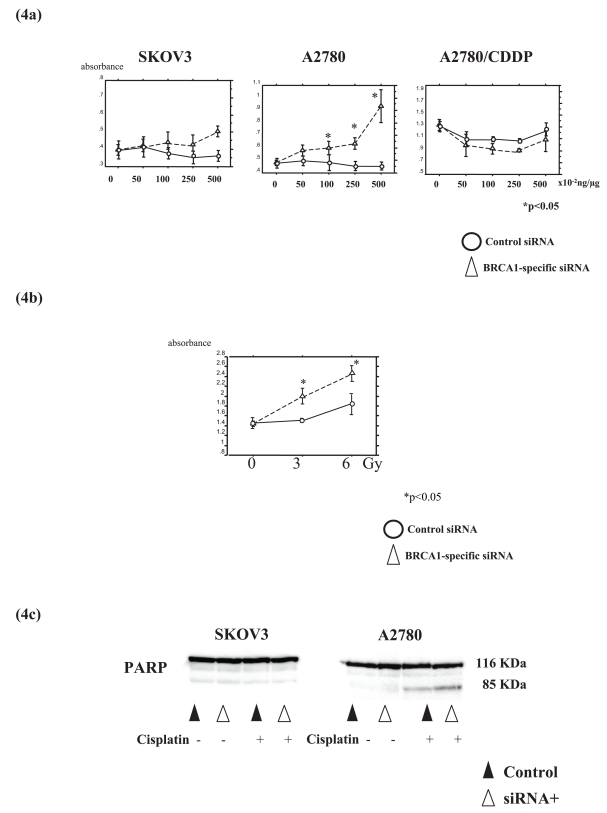
BRCA1 suppression increases the apoptosis of ovarian cancer cells under cisplatin treatment or under irradiation Apostrand assay shows that cisplatin-induced apoptosis were significantly increased in BRCA1-siRNA-transfected A2780 cells (a). After exposure to 3 and 6 Gy irradiation, the increase in apoptosis was observed in BRCA1-siRNA-transfected A2780 cells, but not in other cells (b). PARP cleavage assay (c) demonstrating the presence of the cleaved PARP *M*_r_ 85,000 fragment indicative of apoptosis in A2780 cells transfected with the BRCA1-specific siRNA compared with A2780 cells. In contrast, the SKOV3 cells fail to undergo PARP cleavage under cisplatin treatment.

Then, we examined whether the increased chemosensitivity by BRCA1-siRNA transfection was due to activation of apoptosis. Suppression of BRCA1 in A2780 cells resulted in a dramatic increase in the apoptotic cells under treatment with cisplatin in a dose-dependent manner (Fig. 4a). In contrast, BRCA1-siRNA transfection into A2780/CDDP and SKOV3 cells did not show a statistically significant difference in the apoptosis (Fig. 4a). Exposure to 3 and 6 Gy irradiation also resulted in a significant increase in the apoptotic cells in BRCA1-siRNA-transfected A2780 cells (Fig. 4b). The band density of the PARP *M*_r_ 85,000 product cleaved from the *M*_r_ 116,000 PARP, which is an early hallmark of apoptosis, was 1.9 fold increased in BRCA1-siRNA-transfected A2780 cells under cisplatin treatment (Fig. 4c).

### Increase of apoptosis is associated with up-regulation of p53 and p21

**Figure 5:**
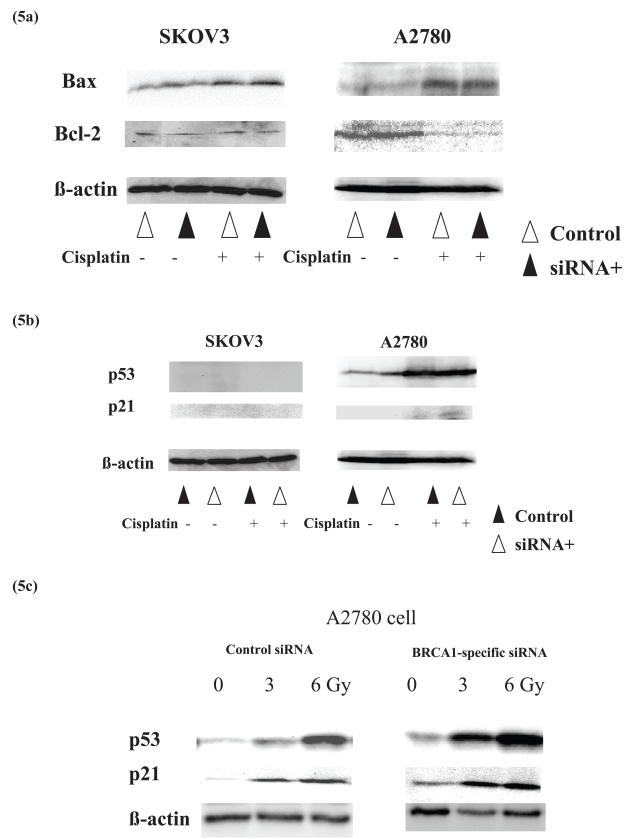
BRCA1 suppression increases the expression of p53 and p21 under cisplatin treatment in ovarian cancer cells. Among the apoptosis-related molecules, expression of both Bax and Bcl2 is not changed between BRCA1-siRNA-transfected cells and control cells under cisplatin treatment (a). However, expression of p53 and p21 is increased under cisplatin treatment in the BRCA1-siRNA-transfected A2780 cells (b). Increase in p53 and p21 in BRCA1-siRNA-transfected cells is also observed under irradiation (c).

Finally, we analyzed the expression of the apoptosis-related gene products, p53, p21, Bax, and Bcl-2. Neither Bax nor Bcl-2 expression was not different between BRCA1-siRNA-transfected A2780 cells and control cells (Fig. 5a). In contrast, the increased expression of p53 and p21 under cisplatin treatment was stronger in BRCA1-siRNA-transfected A2780 cells than control cells (Fig. 5b). After the irradiation, up-regulation of p53 and p21 was stronger in BRCA1-siRNA-transfected A2780 cells than in control cells (Fig. 5c): for p53 increase in 3.3 and 1.8 fold at 3 and 6 Gy, respectively, and for p21 increase in 2.0 and 1.5 fold at 3 and 6 Gy, respectively.

## Discussion

Our previous study on 76 patients with ovarian carcinoma treated during the period of cisplatin-based chemotherapy before the introduction of paclitaxel revealed that there was a tendency towards a poorer prognosis in advanced stage patients with positive BRCA1 expression, i.e., overall survival was 17.7 ± 8.9 months for BRCA1-positive versus 31.6 ± 25.9 months for BRCA1-negative patients (Wang et al. 2004). There have also been reports on the better survival in patients with BRCA1-negative tumor compared with those with BRCA1-positive one, as exemplified in breast and lung carcinomas (Rubin 1996; Ben David et al. 2002; Taron et al. 2004). These in vivo data prompted us to analyze the role of BRCA1 in sensitivity to chemotherapeutic agents. In this study, we examined the chemosensitivity under suppression of endogenous BRCA1 expression using BRCA1-specific siRNA in ovarian cancer cell lines. Our results showed that the sensitivity to cisplatin was significantly increased by BRCA1 suppression in A2780 ovarian cancer cells. The effect of BRCA1 suppression on the chemosensitivity has previously been studied mainly in breast cancer cell lines. Breast cancer cells carrying wild-type BRCA1, such as MCF-7 and MDA-MB231, were more resistant to cisplatin than BRCA1-mutant HCC1937 cells (Tassone et al. 2003). In BRCA1-mutant breast cancer cells, reconstitution of BRCA1 resulted in the cisplatin resistance (Quinn JE et al. 2003). Husain et al. (1998) demonstrated that acquisition of further resistance to cisplatin was associated with an increase in the expression of BRCA1 in MCF-7 cells. In a previous report on human ovarian cancer cells, up-regulation of BRCA1 was associated with more resistance to cisplatin, and the suppression of BRCA1 using its antisense oligonucleotide restored cisplatin sensitivity (Husain et al. 1998). All of these findings indicate that BRCA1 status is very important in the sensitivity to cisplatin in various cancer cells.

More interesting issue is the role of BRCA1 on the sensitivity to paclitaxel. In our study, suppression of BRCA1 did not show the significant changes in the sensitivity in all of the 3 ovarian cancer cell lines. Cisplatin is an intra-strand DNA cross-linking agent that forms DNA adducts, and also causes double-stranded DNA breaks by introducing inter-strand links (Metzler 1986), whereas paclitaxel is an anti-microtubule agent disrupting the mitotic spindle. Therefore, the discrepancy in the effect of BRCA1 suppression between cisplatin and paclitaxel is possibly due to their differences in the signal pathways for the chemotherapeutic effect (Kennedy et al. 2004). This is also supported by the fact that BRCA1 suppression also resulted in an increase in the sensitivity to ionizing radiation, which also induces DNA damages including double-strand breaks. Previous in vitro experiments using breast cancer cells showed that BRCA1-negative cells were less sensitive to paxlitaxel than BRCA1-positive cells (Tassone et al. 2003), and that BRCA1 might be required for the induction of apoptosis in response to paclitaxel in breast cancer cells (Quinn et al. 2003). However, a clinical study showed that locally advanced breast carcinomas negative for BRCA1 expression responded well to taxens, compared with those positive for BRCA1 (Egawa et al. 2003). In mouse ovarian cancer cells, Sylvain et al. (2002) demonstrated that suppression of BRCA1 using the truncated mutant increased the paclitaxel sensitivity. Zhou et al. (2003) reported that BRCA1 mutation in human ovarian cancer cell line increased the sensitivity to paclitaxel. Although we did not find the significant changes in the paclitaxel sensitivity under suppression of BRCA1, further studies are needed to clarify the actual role of BRCA1 in the response to taxens in ovarian carcinoma cells.

BRCA1 has also been reported to contribute to the maintenance of genome integrity. BRCA1 deficiency increases the mutation rate of gene such as p53 (Deng and Wang 2003). BRCA1 was also shown to bind directly to p53 (Derbyshire et al. 2002; Chai et al. 1999), and to enhance trans-activating activity and stability of p53 (Somasundaram et al. 1999; Fabbro et al. 2004). These findings prompted us to analyze the effect of BRCA1 suppression on the chemosensitivity and its relevance to the p53 status. In this study, the increased sensitivity to cisplatin by BRCA1 suppression was observed in p53-wild A2780 cells, but not in p53-mutated A2780/CDDP cells nor in p53-deleted SKOV3 cells. This seem to be consistent with that BRCA1-knockout mice die in early gestation, whereas additional loss of p53 function is able to rescue the same mice (Hakem et al. 1997). Therefore, normal p53 function may be an essential component for induction of cell death due to DNA damage by cisplatin treatment and irradiation. Our findings suggest that BRCA1 cooperates with p53 in the response to DNA damage in ovarian cancer cells. However, BRCA1-negative ovarian carcinomas frequently show p53 overexpression suggesting p53-mutation (Wang et al. 2003). Further studies are needed to verify the role of p53 in BRCA1-dependent chemosensitivity in ovarian cancer cells.

Chemotherapeutic effect is usually influenced by the cell proliferation and the cell cycle, and apoptosis is the final common pathway of cell death due to chemotherapy and irradiation. Our study showed that BRCA1 suppression affected neither the cell proliferation nor the cell cycle of ovarian cancer cells. However, increased induction of apoptosis was linked to the increased sensitivity to cisplatin and to irradiation under BRCA1 suppression in the A2780 cells. Then, we analyzed which pathway is involved in the apoptosis in BRCA1 suppression. Among the apoptosis-related molecules, BRCA1 suppression along with cisplatin treatment did not change the expression levels of Bax and Bcl-2, but up-regulated both p53 and p21 expression in p53-wild A2780 cells. It has also been reported that BRCA1-knockout embryos die in early development being associated with elevated expression of p21 (Hakem et al. 1996, 1998). These findings suggest that p53–p21 pathway plays an important role in the apoptotic cell death due to BRCA1 suppression. Although it has been known that p53 is involved in the induction of p21(WAF1/Cip1)(Zuo et al. 1998), this is the first report on the relevance of p53 and p21 to the chemosensitivity in BRCA1-suppressed ovarian cancer cells. Interestingly, we recently found that activated form of MAP kinase (ERK) was decreased in BRCA1-suppressed ovarian carcinoma cells (data not shown), and this may be consistent with that activated ERK is reported to work as anti-apoptotic signal (Ohmichi et al. 2005).

In summary, the suppression of BRCA1 expression enhances the cisplatin sensitivity and induces apoptosis possibly via the p53–p21 pathway. In contrast, it does not affect the sensitivity to paxlitaxel. Accordingly, the expression of BRCA1 might be an important biomarker for the sensitivity to chemotherapeutic agents. Prospective studies are needed to verify its predictive value in the chemotherapy for ovarian carcinoma patients.

## Figures and Tables

**Table 1:** BRCA1 suppression does not affect the cell cycle of ovarian cancer cells.

	A2780		A2780/CDDP		SKOV3	
	control	BRCA1 si	control	BRCA1 si	control	BRCA1 si
G1	52.57±5.11%	50.03±4.88%	48.87±4.76%	44.77±5.71%	45.07±4.1%	43.03±2.71%
S	16.43±3.18%	19.33±2.81%	10.89±2.18%	10.40±3.23%	9.43±5.18%	9.39±1.86%
G2/M	19.73±2.50%	21.37±2.76%	28.83±2.44%	29.39±3.50%	23.03±1.50%	23.99±2.90%

Values indicate means ±SE.

## References

[b1-bmi-2006-049] Baldwin RL Nemeth E Tran H 2000 BRCA1 promoter region hypermethylation in ovarian carcinoma: a population-based study Cancer Res 60 5329 33 11034065

[b2-bmi-2006-049] Ben David Y Chetrit A Hirsh-Yechezkel G 2002 National Israeli Study of Ovarian Cancer. Effect of BRCA mutations on the length of survival in epithelial ovarian tumors J Clin Oncol 20 463 6 11786575 10.1200/JCO.2002.20.2.463

[b3-bmi-2006-049] Berchuck A Heron KA Carney ME 1998 Frequency of germline and somatic BRCA1 mutations in ovarian cancer Clin Cancer Res 4 2433 7 9796975

[b4-bmi-2006-049] Boyd J Sonoda Y Federici MG 2000 Clinicopathologic features of BRCA-linked and sporadic ovarian cancer JAMA 283 2260 5 10807385 10.1001/jama.283.17.2260

[b5-bmi-2006-049] Catteau A Harris WH Xu CF 1999 Methylation of the BRCA1 promoter region in sporadic breast and ovarian cancer: correlation with disease characteristics Oncogene 18 1957 65 10208417 10.1038/sj.onc.1202509

[b6-bmi-2006-049] Chai YL Cui J Shao N 1999 The second BRCT domain of BRCA1 proteins interacts with p53 and stimulates transcription from the p21WAF1/CIP1 promoter Oncogene 18 263 8 9926942 10.1038/sj.onc.1202323

[b7-bmi-2006-049] Deng CX Wang RH 2003 Roles of BRCA1 in DNA damage repair: a link between development and cancer Hum Mol Genet 12 R113 23 12668603 10.1093/hmg/ddg082

[b8-bmi-2006-049] Derbyshire DJ Basu BP Serpell LC 2002 Crystal structure of human 53BP1 BRCT domains bound to p53 tumour suppressor EMBO J 21 3863 72 12110597 10.1093/emboj/cdf383 PMC126127

[b9-bmi-2006-049] Egawa C Motomura K Miyoshi Y 2003 Increased expression of BRCA1 mRNA predicts favorable response to anthracycline-containing chemotherapy in breast cancers Breast Cancer Res Treat 78 45 50 12611456 10.1023/a:1022101310500

[b10-bmi-2006-049] Esteller M Silva JM Dominguez G 2000 Promoter hypermethylation and BRCA1 inactivation in sporadic breast and ovarian tumors J Natl Cancer Inst 92 564 9 10749912 10.1093/jnci/92.7.564

[b11-bmi-2006-049] Fabbro M Savage K Hobson K 2004 BRCA1-BARD1 complexes are required for p53Ser-15 phosphorylation and a G1/S arrest following ionizing radiation-induced DNA damage J Biol Chem 279 31251 8 15159397 10.1074/jbc.M405372200

[b12-bmi-2006-049] Futreal PA Liu Q Shattuck-Eidens D 1994 BRCA1 mutations in primary breast and ovarian carcinomas Science 266 120 2 7939630 10.1126/science.7939630

[b13-bmi-2006-049] Geisler JP Hatterman-Zogg MA Rathe JA 2002 Frequency of BRCA1 dysfunction in ovarian cancer J Natl Cancer Inst 94 61 7 11773283 10.1093/jnci/94.1.61

[b14-bmi-2006-049] Hakem R de la Pompa JL Sirard C 1996 The tumor suppressor gene BRCA1 is required for embryonic cellular proliferation in the mouse Cell 85 1009 23 8674108 10.1016/s0092-8674(00)81302-1

[b15-bmi-2006-049] Hakem R de la Pompa JL Elia A 1997 Partial rescue of BRCA1 (5–6) early embryonic lethality by p53 or p21 null mutation Nat Genet 16 298 302 9207798 10.1038/ng0797-298

[b16-bmi-2006-049] Hakem R de la Pompa JL Mak TW 1998 Developmental studies of BRCA1 and BRCA2 knock-out mice J Mammary Gland Biol Neoplasia 3 431 45 10819537 10.1023/a:1018792200700

[b17-bmi-2006-049] Horiuchi A Imai T Wang C 2003 Up-regulation of small GTPases, RhoA and RhoC, is associated with tumor progression in ovarian carcinoma Lab Invest 83 861 70 12808121 10.1097/01.lab.0000073128.16098.31

[b18-bmi-2006-049] Husain A He G Venkatraman ES 1998 BRCA1 up-regulation is associated with repair-mediated resistance to cis-diamminedichlor oplatinum(II) Cancer Res 58 1120 3 9515792

[b19-bmi-2006-049] Kennedy RD Quinn JE Mullan PB 2004 The role of BRCA1 in the cellular response to chemotherapy J Natl Cancer Inst 96 1659 68 15547178 10.1093/jnci/djh312

[b20-bmi-2006-049] Merajver SD Pham TM Caduff RF 1995 Somatic mutations in the BRCA1 gene in sporadic ovarian tumours Nat Genet 9 439 43 7795652 10.1038/ng0495-439

[b21-bmi-2006-049] Metzler M 1986 DNA adducts of medicinal drugs: some selected examples J Cancer Res Clin Oncol 112 210 5 3097024 10.1007/BF00395914 PMC12253003

[b22-bmi-2006-049] Miki Y Swensen J Shattuck-Eidens D 1994 A strong candidate for the breast and ovarian cancer susceptibility gene BRCA1 Science 266 66 71 7545954 10.1126/science.7545954

[b23-bmi-2006-049] Ohmichi M Hayakawa J Tasaka K 2005 Mechanisms of platinum drug resistance Trends Pharmacol Sci 26 113 6 15749154 10.1016/j.tips.2005.01.002

[b24-bmi-2006-049] Ozols RF Rubin SC Thomas GM Hoskins WJ Perez CA Young RC 2000 Epithelial ovarian cancer Principles and Practice of Gynecologic Oncology Lippincott Williams and Wilkins Philadelphia 981 1057

[b25-bmi-2006-049] Quinn JE Kennedy RD Mullan PB 2003 BRCA1 functions as a differential modulator of chemotherapy-induced apoptosis Cancer Res 63 6221 8 14559807

[b26-bmi-2006-049] Rubin SC Benjamin I Behbakht K 1996 Clinical and pathological features of ovarian cancer in women with germ-line mutations of BRCA1 N Engl J Med 335 1413 6 8875917 10.1056/NEJM199611073351901

[b27-bmi-2006-049] Russell PA Pharoah PD De Foy K 2000 Frequent loss of BRCA1 mRNA and protein expression in sporadic ovarian cancers Int J Cancer 87 317 21 10897034 10.1002/1097-0215(20000801)87:3<317::aid-ijc2>3.0.co;2-b

[b28-bmi-2006-049] Scully R Livingston DM 2000 In search of the tumour-suppressor functions of BRCA1 and BRCA2 Nature 408 429 32 11100717 10.1038/35044000 PMC2981135

[b29-bmi-2006-049] Somasundaram K MacLachlan TK Burns TF 1999 BRCA1 signals ARF-dependent stabilization and coactivation of p53 Oncogene 18 6605 14 10597265 10.1038/sj.onc.1203284

[b30-bmi-2006-049] Sylvain V Lafarge S Bignon YJ 2002 Dominant-negative activity of a BRCA1 truncation mutant: effects on proliferation, tumorigenicity in vivo, and chemosensitivity in a mouse ovarian cancer cell line Int J Oncol 20 845 53 11894135

[b31-bmi-2006-049] Taron M Rosell R Felip E 2004 BRCA1 mRNA expression levels as an indicator of chemoresistance in lung cancer Hum Mol Genet 13 2443 9 15317748 10.1093/hmg/ddh260

[b32-bmi-2006-049] Tassone P Tagliaferri P Perricelli A 2003 BRCA1 expression modulates chemosensitivity of BRCA1-defective HCC1937 human breast cancer cells Br J Cancer 88 1285 91 12698198 10.1038/sj.bjc.6600859 PMC2747554

[b33-bmi-2006-049] Thompson ME Jensen RA Obermiller PS 1995 Decreased expression of BRCA1 accelerates growth and is often present during sporadic breast cancer progression Nat Genet 9 444 50 7795653 10.1038/ng0495-444

[b34-bmi-2006-049] Tsuruo T Hamilton TC Louie KG 1986 Collateral susceptibility of adriamycin-, melphalan- and cisplatin-resistant human ovarian tumor cells to bleomycin Jpn J Cancer Res 77 941 5 2429947

[b35-bmi-2006-049] Wang C Horiuchi A Imai T 2004 Expression of BRCA1 protein in benign, borderline, and malignant epithelial ovarian neoplasms and its relationship to methylation and allelic loss of the BRCA1 gene J Pathol 202 215 23 14743504 10.1002/path.1507

[b36-bmi-2006-049] Wang Q Zhang H Fishel R 2000 BRCA1 and cell signaling Oncogene 19 6152 8 11156529 10.1038/sj.onc.1203974

[b37-bmi-2006-049] Zheng W Luo F Lu JJ 2000 Reduction of BRCA1 expression in sporadic ovarian cancer Gynecol Oncol 76 294 300 10684699 10.1006/gyno.1999.5664

[b38-bmi-2006-049] Zhou C Smith JL Liu J 2003 Role of BRCA1 in cellular resistance to paclitaxel and ionizing radiation in an ovarian cancer cell line carrying a defective BRCA1 Oncogene 22 2396 404 12717416 10.1038/sj.onc.1206319

[b39-bmi-2006-049] Zuo Z Dean NM Honkanen RE 1998 Serine/threonine protein phosphatase type 5 acts upstream of p53 to regulate the induction of p21(WAF1/Cip1) and mediate growth arrest J Biol Chem 273 12250 8 9575175 10.1074/jbc.273.20.12250

